# Radical Technology Inquirer: a methodology for holistic, transparent and participatory technology foresight

**DOI:** 10.1186/s40309-022-00206-6

**Published:** 2022-07-14

**Authors:** Risto Linturi, Maria Höyssä, Osmo Kuusi, Ville Vähämäki

**Affiliations:** 1 R. Linturi Plc., 00870 Helsinki, Finland; 2 Finland Futures Research Centre (FFRC), University of Turku, Turku, Finland; 3 What Futures Inc., Finland Futures Research Centre (FFRC), University of Turku, Turku, Finland; 4 Parliament of Finland, Helsinki, Finland

**Keywords:** Technology foresight, Radical innovation, Socio-technical transition, Technology generalisation, Value-producing network, Anticipated radical technology

## Abstract

This paper introduces and motivates the Radical Technology Inquirer (RTI) methodology for anticipation of technological breakthroughs and their combined cross-sectoral and social impacts. The primary use of the methodology is long-term policy evaluation and design. The first version of the methodology was published in 2013. This paper reports the current RTI 2018 version, which is based on systematic collection of scientific and technological news and grounded on theory. It combines societal functions with technological opportunities by conceptualising 20 “global value-producing networks” GVNs and 100 “anticipated radical technologies” ARTs. The RTI methodology is participatory, using continuous crowdsourcing and stakeholder evaluations. Each GVN is characterised by a need and an existing and a novel way of satisfying that need and organising the societal function. The methodology combines existing and new foresight methods and concepts to achieve a holistic and transparent approach for anticipating technology-enabled transformative socio-technical developments of the next 20 years. In this anticipation effort, the focus is more on recent weak signals of emerging technological capabilities than on past strong signals, e.g. the diffusion of various technologies.

## Introduction

This paper introduces a novel methodology for anticipating transformations driven by radical technologies, such as artificial intelligence (AI), plasmonics, CRISPR-Cas9, 3D printing and carbon nanotubes. The paper shows that such a methodology can be realised as a novel combination of foresight methods. The purpose is to enable understanding on which established means satisfying common human interests, such as health care, learning or mobility, are going to be revolutionised during the next 20 years and why. A crucial underlying insight is that the technological component of such revolutions often emerges from a field or sector that has not previously played a key role in serving that need. For example, passenger transport has been based on privately owned vehicles operated by a driver. The emergence of electric cars combined with mobile robotics seems to boost the revolutionising of passenger transport towards service with autonomous vehicles. Importantly, digitalisation and automation of vehicles have its origins in the IT industry, from where the automation-increasing service concepts are spreading to other industries.

Many authors have stressed the systemic interpretation of foresight (e.g. [2, 55, 60]). For more systemic interpretation, Dufva et al. [9] have suggested three complementary facets to foresight: knowledge creation and diffusion, enhancing relations and networking and development of capabilities. These three aspects of systemic foresight are relevant both for short-term and long-term technology foresight, but their content and relative importance differ. The methodology presented in this paper contributes especially to knowledge creation on possible future developments.

The methodology presented in this paper anticipates radical technological breakthroughs and their socio-technical impacts on a 20-year perspective. The key argument of the paper is that when the goal is long-term foresight, sector-specific technological foresight must account for the cross-sectoral nature of global sociotechnological development and cover all radical technologies globally in order to be credible. Subsequently, concerning the first facet presented by Dufva et al. [9], knowledge creation should focus on early signals of radical technological breakthroughs impacting on many emerging socio-technical systems. In this early stage, it is possible to anticipate just crude basic features of changes in those global systems, based on noticed evidence of emerging technologies and social functions that these technologies could relate to. In the present paper, the frame of Stiglitz et al. [63] is used to identify these social functions.

Concerning the second and third facet presented by Dufva et al. [9], this paper shows that the knowledge created by the proposed methodology can and has been used to promote networking among actors with the potential to be involved in socio-technical change and to recognise new skills and jobs required by anticipated transformations.

A methodology for cross-sectoral anticipation is particularly useful for decision-makers and designers of public services because they need a holistic understanding of the possibilities created by simultaneous development of various kinds of radical technologies in order to create policies, regulation and infrastructure that enable societally beneficial outcomes and seek to prevent harmful or undesired ones. For example, it is important to consider the development of radical transport concepts together with the design of city infrastructure, given that future cars may spend less time parked but may need recharging and function as distributed energy storage when parked. A methodology to support interpretation is needed, because people tend to make sense of the possibilities of novel situations [72] and radical technologies [62] exclusively from one’s own, experience-based perspective and fail to see the situation from a broader perspective. Classified with Porter’s [56] foresight typology, the proposed methodology has an extrapolative motivation, considers technology and innovation as the drivers and has a scope of multiple technologies and wide-range planning, global locus, longtime horizon, intermediate level of participation and study duration of years.

The present paper proceeds from the observation that existing technology foresight approaches do not provide public decision-makers with a holistic overview of future possibilities brought about by radical developments across all fields of technological development. The traditional diffusion models of technologies used in forecasting (e.g. [48]) are not suitable for long-run anticipation of socio-technical transitions, as these implicitly assume that the innovation adopted by the first individuals or firms does not change much during its diffusion process. The various technology roadmapping approaches, in turn, are best applied in the context of strategic foresight of individual organisations or, in some cases, sectors [54] rather than in the design of public policies, services and infrastructure. The TechCast project [20, 21] has a broad scope and is as such informative for decision-makers. Its methodology is, however, based on subjective expert judgements concerning future proceeding of single technologies in S-curves. This methodology is not able to account for the interaction between different technologies in a transparent and systematic way. This lack of systemic wholeness is a big weakness especially from the point of view of policymakers that make infrastructure-related choices. It can also poorly consider the inertia and interests of present-day stakeholders as opposed to those of proponents of novel technologies.

The methodology introduced, theoretically motivated and illustrated in this paper is called Radical Technology Inquirer (RTI). It is a cross-disciplinary and holistic methodology of technology foresight. It is transparent in the sense that it enables the observation, at every step, of how technologies and their future impacts are identified and evaluated. This means that one can in principle replicate the study, starting from the empirical material. In addition, RTI is participatory in the sense that the gathering and interpreting the significance of the material are crowdsourced, and the credibility of the interpretations is checked in stakeholder seminars. The openness of the developer community of the methodology enables the cross-disciplinary nature of the foresight.

The development of the RTI methodology was originally commissioned from the first and third authors by the Committee for the Future of the Parliament of Finland in 2012. The intention was to provide a robust understanding of the potential societal impact of rising radical technologies. The first Finnish version of the RTI methodology, reporting developments in 100 technology areas, was published in 2013 (from now on “RTI 2013”) ([36]; English edition: [37]). An introduction of this early version of the methodology also appeared in the *European Journal of Futures Research* [68]. In an update report ([38]; in English [39]), new niche innovations were introduced, a participatory methodology of data gathering was fully utilised, a proposal was made for basing the understanding the dynamics of transformation on the regime model [13, 14] and a follow-up was done on how the anticipated technology breakthroughs had evolved since 2013.

In summer 2017, the European Commission started a project (RIBRI) related to Horizon 2020 to “Europeanise” RTI. It took the RTI 2013 as a starting point but modified the methods to serve different aims. Meanwhile, an updated RTI approach was published in 2018 (hereafter “RTI 2018”) [40, 41], but it had not been available when the main empirical material of RIBRI was collected in autumn 2017 and in spring 2018. RIBRI’s final report “100 Radical Innovation Breakthroughs for the future. The Radical Innovation Breakthrough Inquirer (RIBRI)” was published in June 2019 [71]. This article focuses on the detailed description and theoretical motivation of the choices made in RTI 2018 and leaves the interesting comparison with the RIBRI tool to some other connection. However, Kuusi [28] made in Finnish a preliminary comparison of RIBRI and RTI 2018 from the perspective of the future of work.

The most important differences between RTI 2013 and RTI 2018 are the following. The renewed version gives transformations a theoretically grounded structure by anchoring them to the concept of socio-technical *regime* [13, 14]. In the RTI 2018, interests of typical regime actors were systematically identified and needed capabilities, and other requirements were described for each transformation. Also, the definition of technology categories was systematised, and their evaluation methodology was completely redesigned.

The article proceeds as follows: “Theoretical foundation” presents the theoretical foundation behind the key concepts and methodical steps of RTI 2018. “Key methodological elements of the RTI methodology” describes the RTI 2018 tool in sufficient detail to enable replication of the methodology. “The uses of RTI results” shows how the results of the methodology have been applied in policy, educational and infrastructure planning contexts. “Discussion” discusses the limitations and further development possibilities of the methodology, and “Conclusions” states the contributions.

### Theoretical foundation

The starting point of the RTI methodology is empirical. It begins with systematic horizon scanning for published news on technological advances. To manage the observing and grouping of evidence of all kinds of radical technological developments, the concept of “anticipated radical technology” (ART) is introduced. ART refers to *the transformative promise of a cluster of technological niche innovations* or social innovations based on technological opportunities. Transformative promise means that an ART has unrealised potential for applications that are anticipated to have radical impacts on current socio-economic structures or behaviour.

In RTI, all radical technological developments globally are divided into 100 ARTs. A specific ART is not limited to a specific technological solution but includes any technological solution that promises to fulfil a general goal, such as “autonomous cars and trucks” or “antibacterial and repellent surfaces”. Thus, an ART should be understood as a cluster of rapidly advancing technological solutions potentially enabling the same function or a possibly opening bottleneck (comp [5, 58].), in other words, a trajectory from a problem area towards its possible solution, with batteries in electric cars being a good example. For instance, the RTI report’s description of ART no. 17, “easy 3D printing of parts”, shows that 3D printing is already possible, but it is not yet easy enough to be commonplace.

Ideally, the solving of an ART bottleneck(s) directly enables new applications. Even when the functions are similar, like solar panels and fuel cells, which both produce electricity, they form separate ARTs if the respective bottlenecks differ greatly from each other. Typically, ARTs are systems with many bottlenecks whose solutions may depend on each other. A driverless car is a good example. It is defined as an ART, but many of its potential component technologies are also defined as separate ARTs as they enable several other applications besides driverless cars.

As an ART matures, some of the solutions typically become dominant designs [1], while the development of others stalls or ceases altogether. A related recent concept is “next technology” (NT) that also refers to technologies (e.g. AI) with potential transformative impacts (e.g. [17, 59]). Evidence of advancement of ARTs can be interpreted as indicators of future capabilities. In this paper, such interpretations are defined weak signals [10].

In RTI 2018, special attention is paid on assessing the cumulative future impact of ARTs on their socio-technical contexts. The methodology is inspired by Toffler’s [66] waves that show societal and technical developments as interconnected, whereupon the anticipation of technological advances is considered a fruitful perspective to the anticipation of potential societal changes, too. To this end, the present paper introduces the concept of the global value-producing network (GVN). It is a future-oriented concept that, in addition to related emerging technologies and their regulation, is based on social functions.

RTI 2018 recognises altogether 20 GVNs, such as passenger transport, redressing disabilities, producing experiences and power structures. The GVNs promote “social needs” or social functions that are closely linked to basic dimensions of human well-being. The Commission on the Measurement of Economic Performance and Social Progress [63] suggested for measuring the following social functions that promote human well-being: material living standards (income, consumption and wealth), health, education, personal activities including work, political voice and governance, social connections and relationships, environment (present and future conditions) and insecurity, of an economic as well as a physical nature. These basic social functions nicely summarise the result of the long discussion concerning dimensions of human well-being and their measurement. This global discussion started already in the 1970s. Especially important was the OECD social indicator programme [34, 50]. The 20 GVNs can be seen as a synthesis of emerging radical technologies and various functions that promote the mentioned eight social functions.

It is essential that the proposed RTI methodology allows flexibility concerning ways that promote social functions. As discussed a further below, every GVN is assumed to include two images of future that are alternative ways to promote respective social functions: a *dominant regime* and an ART-enabled *challenger regime*. The dominant regime is presented as being structured around mature technologies, while the structure of the challenger regime is envisioned around emerging ARTs. As images of future ([25]; comp [23].), these represent a business as usual and an emerging radical change.

**Fig. 1:**
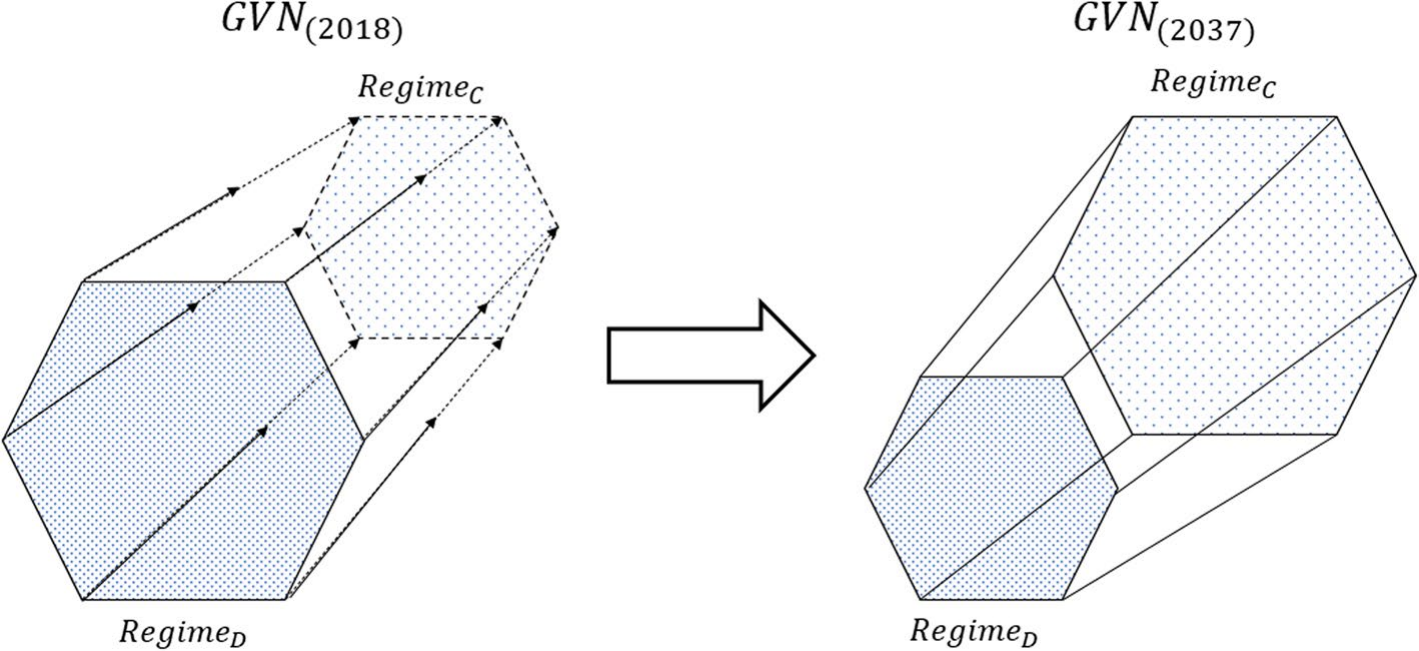
Socio-technical transformation of a GVN (prism shape) involves a shifting balance between the two co-existing RTI regimes as time goes by; the dominant regime (front end) diminishes as the challenger regime (depicted with a dashed line in the back end in 2018) gains ground. Each edge depicts a different change in the regime, such as a change in technology, politics, industry and culture. Compare with the three waves of Alvin Toffler [66]

Both the dominant and challenger regimes fulfil the GVN’s primary goal (such as the transfer of goods), albeit via different socio-technical solutions. GVN transformation is understood as the challenger regime becoming the dominant one (Fig. 1). The regimes are considered to come to co-exist as parts of the GVN by 2037. Both regimes are striving to fulfil the GVN goal via different means and socio-technical networks that possibly but not necessarily remain quite separated from each other.

Transformation towards the challenger regime can be gradual or involve rapid sequences. Different actors perceive it as happening at different times: change in investor behaviour typically happens earlier than change in mainstream consumer behaviour.

Each GVN should be understood as a networked interaction process of all stakeholders involved in fulfilling a specific primary goal that defines the GVN. Organisations and individuals often participate in several different value-producing networks. Hospitals are a good example. They provide housing, security, nourishment, logistics and health services and thus add value to several value-producing networks.

The idea of GVN is strongly inspired also by the multi-level perspective on technological transitions (MLP). The regimes of GVN resemble MLP’s concepts of the socio-technical system and socio-technical regime (e.g. [13, 14]). MLP defines regime as a set of rules, which creates stability to development by affecting the social actors. “Examples of regime rules are cognitive routines and shared beliefs, capabilities and competences, lifestyles and user practices, favourable institutional arrangements and regulations, and legally binding contracts” ([15], p. 27). MLP regimes are conceptualised around social functions or practices such as mobility, sustenance, health care and housing. Also, in RTI, the focus is on social functions that are satisfied in the context of socio-technical regimes.

**Fig. 2:**
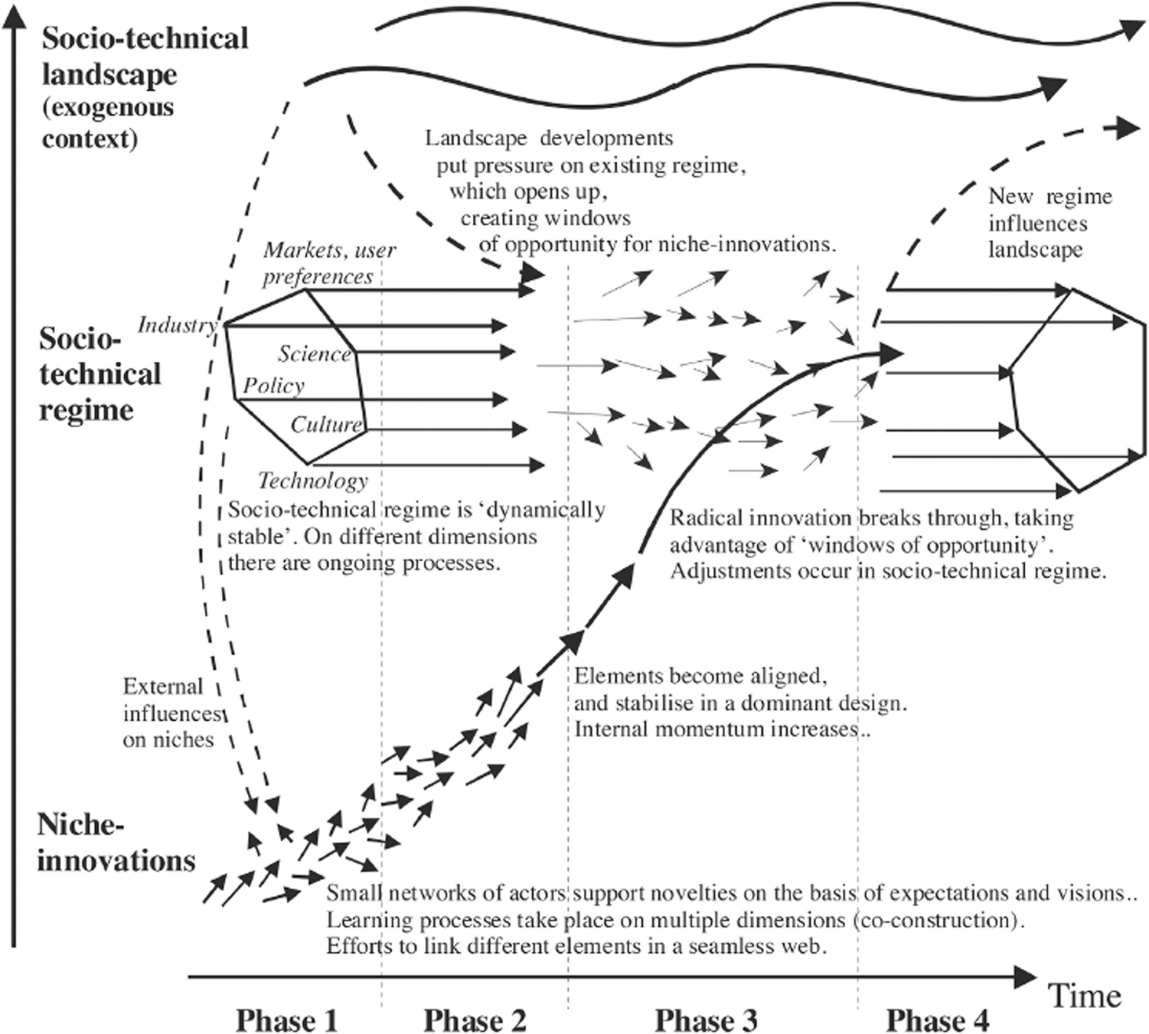
Multi-level perspective on socio-technical transitions ([16], p. 226; reprinted with the permission of the copyright holder)

MLP has become the state-of-the-art way of studying socio-technical change during the past 15 years, especially among those researching so-called *sustainability transitions* [15, 33, 46]. MLP has influenced futures studies as well (e.g. [35, 47, 51, 70]), and its relevance for technology foresight is also recognised (e.g. [54]). MLP is theoretically interesting for technology anticipation. It not only includes technological advances but relates them to a systemic whole by accounting for the social and institutional structures that channel and hinder technological developments. Figure 2 sums up the idea of MLP [16]. It illustrates how emerging niche innovations gradually connect to challenge a prevailing socio-technical regime.

In the RTI context, Fig. 2 is also a way to describe how a recent dominant regime within a global value-producing network (GVN) will transform to or be at least partially replaced by a new challenger regime. In RTI, the emphasis is on assumed co-existence of dominant and challenger regimes. This means that maturing radical technologies are considered as part of emerging alternative systems, being compatible with anticipated novel infrastructural solutions and ways of use. The dominant and the challenger regime share a prime goal; only the socio-technical means differ — private cars with a driver represent the existing dominant regime for transporting people; mobility services with autonomous vehicles represent the envisioned challenger regime for human transport that currently exists only in a pioneering phase. Electric vehicles support the emergence of the challenger regime. Yet they have also become more compatible with the standards, infrastructures and regulations of the dominant regime, meaning that the switching costs are lower and user practices do not have to change too much while adopting the next technology as an alternative to the old one.

Geels has argued that “[p] ositive and negative influences from other regimes on the focal regime are an understudied but promising topic” ([15], 32), and that future development of the MLP model needs to pay greater attention to multiple innovations and system reconfiguration, in particular interactions between niche innovations, adoption of niche innovations in existing systems and interaction between multiple systems [16]. A key argument in this paper is that when the aim of research is turned from analysis of the past to anticipating the future on a 20-year perspective, foresight related to detailed systemic niche interactions is not feasible due to emergent socio-technical structures. Hence, it is important to understand that the ARTs of RTI are described simply in terms of their current maturity level, the technological bottleneck they solve and the societal interest and industry funding they attract in a somewhat comparable manner as Toffler did in his Third Wave [66] — but not in terms of their particular development contexts.

In fact, stepping over micro-level interactions between development contexts enables the holism of RTI. Without such simplification, it would be methodologically impossible to anticipate the development of the socio-technical system — all innovations in all GVNs — globally albeit coarsely.

**Fig. 3:**
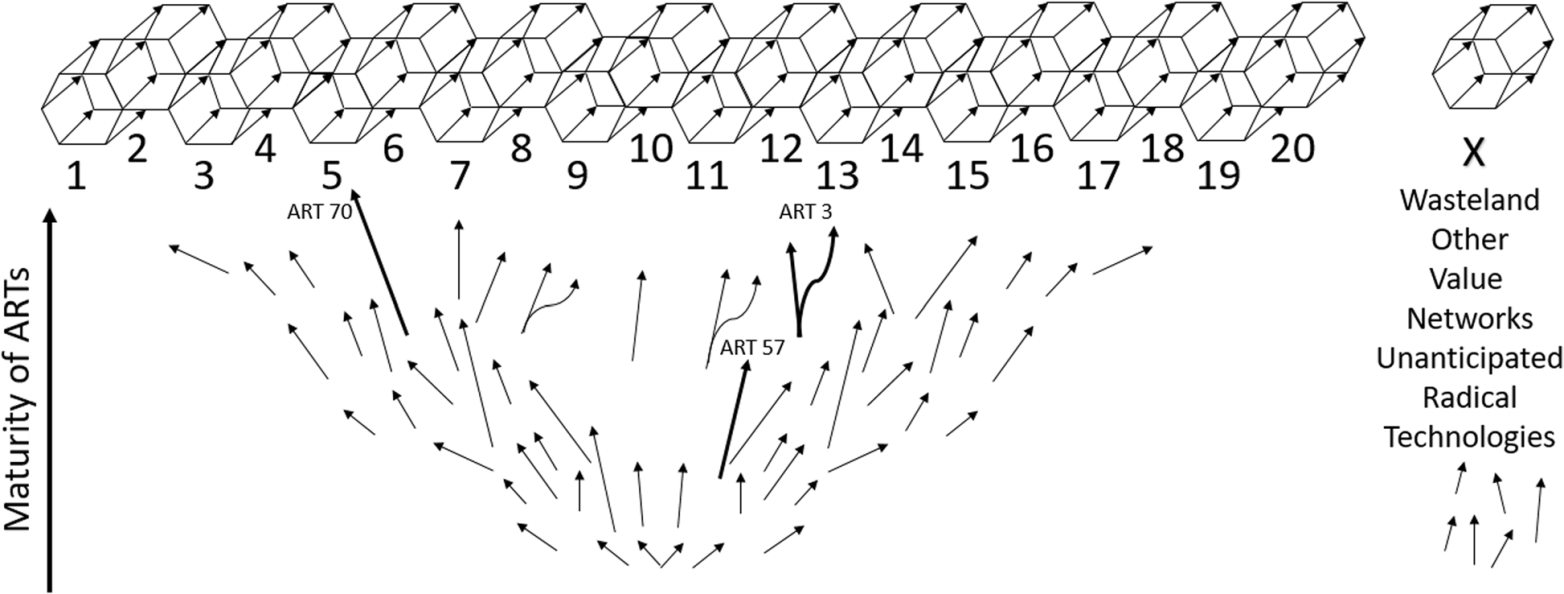
Model of global socio-technical system as 20 global value-producing networks (prisms) and anticipated impacts of 100 anticipated radical technologies (arrows) on them

Figure 3 below illustrates the global RTI perspective. The upper part of the figure depicts the 20 GVNs (for GVN number/topic, see Table 2). It also features a residual hypothetical GVN (Wasteland) to recognise that new human needs could emerge as the challenger regimes advance. For example, a need to digitise human consciousness or to arrange human life on Mars is already conceivable, but there is no evidence of its existence as a major driving factor of technological development. The arrows in Fig. 3 represent the ARTs. Each arrow’s position indicates respective ART’s maturity, length of its development speed and direction of the main benefiting GVN or GVNs. Forked arrows represent ARTs that are developed to serve a function in separate GVNs. The figure is schematic, so most arrows are only illustrative. The three numbered arrows roughly depict the maturity, development speed and main benefiting GVN of actual ARTs: ART 3 personal health diagnostics systems, ART 70 rapid development of photovoltaics and ART 57 radical longevity.

So far, the literature that has discussed transformative changes of socio-technical regimes from an anticipatory point of view has focused on individual rather than multiple socio-technical regimes. Emphasis has been on regimes where the transition is most evident: in the energy system towards renewable energy sources and in the traffic system towards new energy sources and automation [4, 6, 12, 32, 47]. This paper advocates the view that due to path dependence and threshold cost considerations, differences between paces of technological change in different regimes as well as related cross-regime impacts are particularly important to account for when a long-term anticipatory or foresight goal is adopted. If such cross-regime impacts are neglected, sector-specific foresight results may remain seriously biased. The proposed approach proceeds from the observation that investments are first made and technologies developed in the field or sector where the incentives are sufficient, whereas the major transforming impact of the funded innovation may eventually take place in another sector or regime. Typically, development starts when switching costs and quality requirements are low [7] or when they yield high value. A piece of technology becomes adopted and adapted by other sectors or regimes when it reaches a level where the required investments in, e.g. R&D, infrastructure and marketing become sufficiently low through being developed for other uses first. Also, regulatory change, productive capability or other enablers may be promoted for the reasons relevant in one sector, while their impact may be more extensive in another sector. For example, the lithium battery received a major boost from the mobile phone industry before achieving credibility in the automobile industry or more recently as power grid storage in the energy sector.

### Key methodological elements of the RTI methodology

The methodology of RTI 2018 consists of several methodical steps. Some of these are known foresight and future methods; some are novel ways to combine the previous steps (Table 1). The methodology sections below open the RTI methodological steps in more detail.

**Table 1:** RTI methodology steps and forecasting methods used in each step

Methodology step	Used methods	Relevant section of this paper (Reference to studies with comparable interpretation of the method)
Defining ARTs	Horizon scanning for factual evidence for identifying weak signals	This section (Comp. with horizon scanning by volunteers in [8])
Defining the maturity of ARTs	Own criteria	This section (Comp. NASA^a^)
Defining GVNs and respective dominant and challenger regimes	Formulating images of future	This section (Comp [25].)
Qualitative assessment of the impact of ART on GVN	Identifying and assessing weak signals (weak signal as an indicator of a resolving a bottleneck)	This section (Comp [10].)
Quantitative scoring of the weak signals	Assessing the maximum potential impact of the weak signal on the GVN Assessment of the scale of impacts based on question lists	This section (Comp. Tetlock et al., 2015)
Ordering of the ARTs based on scoring and maturity		This section
Iteration	Expert panels	This section (Comp [52].)
Policy recommendations to enable or to prepare for challenger regimes	Requirement-related backcasting	Next section (Comp [18]. )
Follow-up	Comparing the results of RTI 2013 and RTI 2018	Next section

^a^https://www.nasa.gov/directorates/heo/scan/engineering/technology/technology_readiness_level

#### Defining ARTs

For RTI 2013, the body of evidence for technological change was initially gathered by the first and third authors from various technology assessment sources in late 2012 and early 2013. The methodology soon came to incorporate participatory elements especially for horizon scanning and formulating images of the future. For finalising the RTI 2013, a social media group was founded. It soon included hundreds of participants to complement the initial findings of the authors. Since then, the group has grown to include 3400 participants. For RTI 2018, up to 2600 people — about 1000–2000 of them monthly active readers — produced 1000–3000 monthly reactions, c. 200–400 comments and 40–60 separate threads with c. 1–3 useful links each. Each new thread is required to start with a source link describing a novel technology breakthrough or major advance in the maturity of earlier technologies that could be assumed to have a radical impact on at least one GVN. This is followed by discussion on the potential of the presented breakthrough and possibly more useful links.

The final 1600 source links selected by the authors from this crowdsourced original data for the RTI 2018 report were suggested by 213 people from diverse fields and interests, 26 of whom were female. Nearly all have Finnish origins as the working language of the groups is Finnish. However, global content is sought, and the source links are almost completely in English, from c. 400 different global sources. Due to readability and availability, popular science and technology sources have often been preferred after fact-checking, when necessary, over the original scientific publications. Business and technology news are typically used for R&D funding information and assessment of the practical benefits of each advance.

When comparing this extensive crowdsourcing with the initial approach of RTI 2013, two findings are apparent. A small group has a limited view. It is very difficult to find the unexpected due to the fragmented nature of today’s science and technology. There are also breakthroughs which no one even in a larger group would expect. In the RTI approach, this is mostly remedied by constant monitoring of various science news sources as the important breakthroughs appear in daily news streams and are recognised even when unexpected. This kind of continuous crowdsourcing also seems to motivate people to share their findings.

The gathered 1600 articles on scientific and technological breakthroughs belonging to very diverse scientific disciplines had to be organised to assess their impact in an orderly fashion. For RTI 2018, they were initially categorised into 10 technology groups. The process was iterative, and technology group boundaries emerged from the source material itself. Each group was then further divided into what came to be called anticipated radical technologies (ARTs). Each ART was described by detailing the problem area, recent and anticipated development, funding sources and industrial motivations.

The basic requirements for each ART are as follows:

- The ART shows promise based on a recent breakthrough in research or clear signs of new innovations spreading to the market and driving the learning curve.
- The function of the ART is sufficiently defined to be understandable and potentially mature and influential by 2037.
- The potential impacts of an ART can be recognised through crowdsourced reliable science news or stakeholder workshops.

There are altogether 100 ARTs (for example see Table 2). The precise amount of 100 ARTs was chosen for communication purposes, but this scale also enabled finding a place for each piece of evidence in a way that keeps the categories understandable and meaningful and the residual category (no. 101) as insignificant compared to the others. The source links create plausibility to an ART and assist in evaluating its potential.

**Table 2:** Global value-producing networks with their most potential transformations

Global value-producing network (GVN)	GVN’s prime goal and moderating values	Dominant regime characteristics	Challenger regime characteristics
**1. Passenger transport**	Transporting people from one place to another comfortably, safely, cost-effectively and by offering freedom of choice	Private cars operated by a driver, public transport	Autonomous transport as a service
**2. Logistics**	The transfer of goods, equipment, animals, raw materials and waste from one place to another through convenient, accurate, cost-effective and common means	Transport operated by a driver, repetitive automated loading	Autonomous transport, smart loading robotics
**3. Manufacturing of goods**	The availability and manufacturing of physical goods and equipment with available raw materials in a functional, easy, cost-effective and high-quality way	Industrial, centralised, repetitive manufacturing	Robotised, decentralised, discrete manufacturing
**4. Sustenance**	Human sustenance, as well as pet sustenance, i.e. the intake of energy and the necessary nutrients and trace elements in a healthy, secure, economical and enjoyable way	Agriculture, food industry, distribution channels	Urban agriculture, discrete and local robotic food preparation
**5. Energy supply**	Need-based, acceptable and reliable energy for buildings, transportation, machines and processes in a cost-efficient way	Centralised and fossil energy sources, peaking power plants	Renewable, decentralised energy sources and energy storage
**6. Materials**	Cost-effective supply of raw materials and materials used in production of goods, chemical industry and construction with minimal negative impacts and sufficient quality	Mining-based products, energy-heavy process industry	The circular economy, renewable materials
**7. Built environment**	Designing, constructing, maintaining and demolishing spaces and routes with demand-controlled location and conditions for the activities and mobility of people, animals, equipment and plants and providing technical equipment for them cost-efficiently and respecting regulations	Traditional construction and maintenance	Robotised construction and maintenance
**8. Exchange**	Transfer of ownership and access rights reliably, locally and flexibly with the lowest possible search, agreement and delivery costs	Brands, physical retail locations, hierarchies, B2B2C	The reputation economy, e-commerce, P2P, C2B2C
**9. Remote impact**	Easy and safe impact on things and events in places where the impacter itself is not physically present	Telephone, television, internet, social media	VR/AR, avatars and other remote control
**10. Automation of work**	Acceptable and cost-effective replacement of human work with machines that are easy to use and reliable and that create high-quality results	Centralised automation and human-steered machines	Decentralised robotics based on AI and crowdsourcing
**11. Work and income**	Securing own well-being and the well-being of loved ones by deeds that fit one’s own skills and are personally relevant	Salaried employment related to specialisation and exchange	Cooperation, self-sufficiency, micro-entrepreneurs
**12. Health care**	A person living his/her life healthily while the body and mind remain functioning and staying well-being and beautiful as long as possible	Healthcare system, general health recommendations	Self-diagnostics, gamification, individual nutrition
**13. Redressing disabilities**	Compensating functional deficiencies and optimising the functional ability of a person in everyday life with assistive devices and by facilitating the operating environment, taking into account the social costs and benefits	Institutional, outpatient and family care, cheap-assistive devices	Compensating functional deficiencies via robotics, AI, avatars, artificial organs, crowdsourcing
**14. Acquiring information**	Acquiring information perceived as trustworthy and credible about people and things of personal interest	Certified research, reports, news	AI, crowdsourcing, personal instruments and applications
**15. Proficiency and its proof**	Demand controlled proficiency and proficiency demonstrations — emphasising the recognisability of proficiency, sensemaking and procedural and methodological skills	Educational institutions and qualifications, on-the-job learning	Flipped learning and independent learning, AI, proficiency demonstrations
**16. Producing experiences**	Emotional experiences, the joy of insight and shared experiences generated intentionally in different contexts	Focus on producers and consumers, mass entertainment, tourism	Games, shared VR, AR, interaction, AI
**18. Safety and security**	Freedom from an external threat and the opportunity to promote one’s own goals within known and predictable rules that support caution and justice	Material safety in society, social security	Decentralised, individual and crowdsourced safety and security
**18. Collaboration and trust**	Increasing collaboration on issues that produce synergistic benefits by supporting transparency, risk management and trust	Guaranteed by authorities, brands and hierarchies	Peer-to-peer trust through platforms and transparency
**19. Existential meaning**	Experiencing own existence and functions as meaningful, typically through self-realisation, serving others or joining a greater story or mission	Work, position, social network	Achievements, likes, participation, communities
**20. Power structures**	Productive and equal decision-making in collaboration, in public activities	Regional power structure, opaque power	Subject-matter subsidiarity, location independence

The perspective of 20 years was chosen as a suitable time frame for anticipation to enable the subsequent planning and implementation of the legislative changes and public investments that the results seem to call for. Further support for the time frame is gained from research by Pezzoni et al. [53], who show that novel technological ideas seem to realise much of their potential in 20 years after the first patent.

The defining of the ARTs is not absolute but needs to be changed as the problem areas evolve. The importance of each ART depends both on the evaluation of the impact of the ideas, the creativity in coming up with the ideas and the coverage of the impacts of the ART. For example, RTI 2013 included big data as one ART. In RTI 2018, big data had been divided into several more functional ARTs. Essentially, ARTs should be formed around a function that accurately shows where the unrealised potential lays (in the case of big data, the greatest unrealised potential moved from data formation to data processing).

#### Defining the maturity of ARTs

As ARTs are defined as anticipated solutions to bottlenecks, it is essential to assess the probability of opening the bottleneck within the required timeframe. This is accomplished with a stepwise maturity scale where passing each step makes the opening of the full potential more probable. The final step brings the ART to market competition and into the realm of well-known learning curve effects. If successful, this brings the costs down, which is crucial for further bottlenecks to open in related, cost-limited areas or GVNs. Different applications have varying tolerances for cost. For example, solar panels were used in space exploration when the cost of solar panels was still prohibitively high for land-based electricity production. Thus, the last step is never complete. As an ART matures, some of the solutions typically become more dominant, while the development of others stalls or ceases altogether.

The maturity of each ART was evaluated on a seven-level scale:

1. The scientific principle that enables the breakthrough in the defining function has been proven to be possible at the level of a theoretical model, published by a research team from a reliable research institute. The observation is recent, and there appears to be academic interest in the subject.
2. The scientific model enabling the breakthrough in the function-related area of application has been verified with concrete and credible laboratory testing. The research groups are funded, and the academic motivation is clear. Progress appears to be taking place.
3. A laboratory prototype indicates that some material problems previously preventing the breakthrough have been solved, and the necessary functionality has been achieved. Production costs or durability has yet to be solved at this level. The research groups are funded, and the academic or commercial motivation is clear. The progress is recent, and the new opportunities presented are significant.
4. PoC (proof of concept): A functional prototype has been scientifically or commercially demonstrated to meet the requirements of commercialisation in terms of its functionality and viability for production. In terms of its functionality, the prototype exceeds the benefits or involves lower production costs than previous solutions after the economies of scale have been taken into account. The progress is recent.
5. Competition between several well-capitalised market actors developing the production prototype for this ART after the PoC phase, or a production prototype clearly in the finishing phase, with investments made to launch production. The progress is fast paced.
6. Products being delivered to customers in increasing amounts: The economies of scale are expected to reduce prices considerably. The areas of application are expected to expand. R&D activity is at least partly based on internal financing, and the expansion of production has been clearly financed.
7. Competing products are being delivered, and customer demand is on the rise. Competition is internally financed to a significant degree, and growth is financed by major industrial companies, a wide clientele or venture capitalists. The development of product properties involves significant known potential.

Pre-existing scales were studied, but none seemed intended for this kind of long-term evaluation. For instance, the criteria developed by NASA [49] and utilised by for instance the EU Horizon Europe programme^1^ share some of these features, but it does not adequately address the research and market interests or funding issues.

#### Defining GVNs

GVNs represent the demand-pull or interest-bearing side of the RTI methodology, while ARTs describe the potentially opening bottlenecks. When a particular ART is essential for a specific GVN transformation, these may seem two sides of the same coin, but concepts GVNs and ARTs are fundamentally different. The GVN concept is used to organise various stakeholders’ interests^2^ or their potential demands into clusters. Each of these clusters promotes a more general social function or “social need” of Stiglitz et al. [63]. GVNs defined core element is a prime goal, such as “the transfer of goods, equipment, animals, raw materials and waste from one place to another” of the GVN of logistics ([41], p. 88). A GVN’s prime goal may be intrinsic (e.g. sustenance) or instrumental (e.g. logistics). Each GVN is defined to have also important qualitative, moderating values, such as ease, accuracy, cost and prevalence of the transport method in the GVN of logistics. These values are related to the way the goal is met. They are central for analysing the plausibility of transformation of any GVN.

GVNs were defined iteratively. The starting point was an intuitive list of GVNs with specific prime goals, such a s sustenance and transport. Then, the first author initially compared this list with various industrial classifications and “Yellow Pages” types of listings of organisations, including listings of third sector organisations. Each organisation type was categorised into an GVN in terms of what prime goal it mainly serves. For instance, the church was seen as a provider of meaning to life. If no suitable GVN was found, it was created while striving to keep the number of GVNs as low as possible. The GVN list was considered complete when no organisation type could be found whose prime goal would not fit into some of the GVNs. Also, each GVN had to include a significant number of organisation types whose primary goal served the GVN prime goal.

Such category forming is a creative process rather than a formal method. One can, however, evaluate the choices with three criteria: simplicity, consistency and relevance. A key boundary condition concerning the relevance is that the GVNs cover all well-being promoting social functions of Stiglitz et al. [63].

Finally, the dominant regime based on currently dominant technological solutions was described for each GVN. Then, weak signals relating to each GVN were used to characterise the holistic transformative image of future that is the challenger regime. In reality, this required that the weak signals, described below in the subsection “qualitative assessment of the impact of ART on the GVN”, had first been constructed in a crude form. Discussion in the social media group and stakeholder groups played an important role in this (see subsection “Iteration”). Next, the main drivers and tensions between the dominant and challenger regimes of each GVN were specified. Additionally, the plausibility of the challenger regime was considered in relation to the interests and power positions of stakeholders related to both regimes.

In the RTI methodology, a single GVN is considered plausibly defined under the following conditions:

1. There are good reasons to believe that the prime goal of GVN and its moderating values will remain real and pragmatic goals during the next 20 years.
2. All major organised activities can be seen to fit into the defined GVN framework.
3. During the next 20 years, the challenger regime is inclined to increase its plausibility.
4. During the next 20 years, the required enabling ARTs are inclined to increase in maturity.
5. The overall set of GVNs cover all main social prime goals that will be relevant during the next 20 years.
6. No major transformation is plausible, which cannot be explained through some GVN.

There is no perfect solution for dividing stakeholders’ interests or their potential demands into GVNs. Therefore, continuous iteration, described at the end of this section, has been adopted. Iteration has led to the model evolving somewhat from the original RTI 2013. In RTI 2018, each of the 20 GVNs has a separate, clearly defined prime goal that is satisfied by the existing dominant regime but could conceivably also be reached via a challenger regime after the maturation of anticipated radical technologies. Combined, the 20 GVNs seem to cover almost all organised activities focused on the one hand on social functions of Stiglitz et al. [63] and on the other hand on stakeholders’ interests or their potential demands of a typical western society (see Table 2).

#### Qualitative assessment of the impact of ART on GVN

This section shows how the ideas of anticipated radical technologies (ARTs) and the global value-producing networks (GVNs) are used to assess the dynamic relationship between technological and socio-economic development. This requires describing how each specific ART might impact on each GVN. In RTI 2018, these descriptions are short statements, labelled “weak signals”. Starting from Ansoff [3], there are many definitions of weak signal [69]. Dufva [10] gives the following definition that is rather close to our use of the concept: a weak signal is an indicator of a potentially emerging issue that may become significant in the future.

Weak signal statements originate partly from source materials, partly from discussions in the supporting social media group and various stakeholder workshops and seminars. They have been complemented by the first and third authors.

To illustrate, the short statement concerning the possibilities of ART 11 “Speech recognition/synthesis and interpreting” in GVN 2 “logistics” was “A distribution robot can converse with customers (verbot) and act as a shop, for example”. The statement needs to show an ART/GVN pairing having the potential ability to concretely deliver significant instrumental added value to existing practices, for example by saving costs, facilitating people’s everyday lives, increasing comfort or by strengthening or weakening the structures of power.

To give another example, for ART 70 “rapid development of photovoltaics” in GVN 5 “sustenance,” the statement is “The improving efficiency and price of solar panels and LED lights may lead to it becoming sensible to cultivate plants more extensively indoors under LED lighting” ([41], 108).

#### Quantitative scoring of the weak signals

After initial qualitative assessment, for RTI18, the first and third authors of this paper made 2000 numeric evaluations, one for each short statement for the ART/GVN pairing. These preliminary evaluations were reviewed by crowdsourcing and expert panels and then finalised by the two authors. The assessment was made in respect to the goal and the moderating values of each GVN. It resulted in values of 0, 1, 3, 5, 10 or 20. The highly logarithmic scoring emphasises qualitative change, as the values 10 and 20 can only be scored by ARTs that are anticipated to contribute to the transformation of the respective GVN. The scoring process has analogous features with the probability-based scoring of future events in Tetlock and Gardner [64]. The points from 1 to 20 were assigned according to the following, relatively loose criteria^3^:

- One point: the maturation of the ART enables concrete benefits that make it worthwhile to apply the technology to the mentioned use.
- Three points: the maturation of the ART enables tangible value impact on the GVN’s goal. A tangible impact within the developed world would be on average an economic impact at the annual level of €2–20 per person or an impact of 1–10 h change in the time used in the annual everyday life per person.
- Five points: the maturation of the ART enables significant value impact related to the GVN’s goal. A significant impact within the developed world would be on average an economic impact at the annual level of €20–200 per person or an impact of 10–100 h in the annual everyday life per person.
- Ten points: the maturation of the ART *enables*, *as one option*, the challenger regime. On an annual level, the potential impact must exceed €200 per person or influence on a weekly basis the everyday life of at least one in ten people.
- Twenty points: the maturation of the ART is *a necessary, irreplaceable part* of the challenger regime. On an annual level, the potential impact must exceed €200 per person or influence on a weekly basis the everyday life of at least one in ten people.

When it comes to the transformative impact of ARTs, their economic potential is only part of the assessment. Equally important is the anticipated impact of the ART to the everyday life of people. Hence, the alternative assessment in terms of time use is considered equally important. For instance, from the perspective of the GVN of “redressing disabilities”, robotic legs as an alternative to a rollator would enable a person to climb stairs and access rougher terrain. The anticipated impact per person is calculated either through the economic value or by estimating the number of users and the time they would spend on robotic legs in 2037. The larger of the alternative impact types is selected.

Table 3 below gives examples of assessing ARTs from the perspective of GVN “Logistics”. Out of the 100 ARTs, 70 were assessed as being relevant for logistics with a weight of at least 1 point. Out of those 70, the table features two examples from each point category. Examples include also such relevance, which most people would consider having a negative impact.

**Table 3:** Assessment of the relevance of ARTs for achieving the goal and values of the GVN of logistics, ten examples

ART number and name	Short statement of the matured ART’s anticipated relevance for the GVN of logistics	Points
6, environment 3D scanning and positioning	Environment 3D scanning and positioning in relation to the model or absolute positioning are a necessary requirement for autonomous transport	20
16, real-time 3D modelling of environment	A self-driving vehicle absolutely requires a real-time situation picture of its environment and other moving vehicles and people. Detecting and identifying other moving vehicles, people and material are an advantage	20
23, memristors and neural processors	Increasingly small devices have the storage capacity for artificial intelligence that is required for autonomous control, leaving more room for freight. The transport of small packages by quadcopter benefits from this the most	10
39, 3D printing of buildings and constructs	Automation of street and road repairs by 3D-printing structures	1
41, ubiquitous environment and Internet of Things	The unique identity of goods enables crowdsourced freight transport, address changes during transport and right-based key management when goods change hands	10
49, production of nanomaterials	The affordability of sturdy, lightweight materials and efficient batteries is key for aircraft	3
67, LED farming, robotic farming	The needs of food logistics are going to completely transform when cultivation shifts from cyclical and agriculture-dominated cultivation to continuous urban and factory cultivation	5
70, rapid development of photovoltaics	Electric transport benefits from inexpensive solar energy. For example, it may have a material effect on small waterborne vessels and rail transport, at least in southern countries	5
81, power lasers, ray guns, railguns	Ray guns can efficiently disturb traffic	1
99, MyData and GDPR	The transfer of logistics history information (MyData/GDPR) from one operator to another can facilitate logistics	3

Without some relevant tangible evidence, the impact value between an ART and a GVN is evaluated to be zero, and there is not even a weak signal of anticipated competence. An example of an ART deemed irrelevant for the GVN of logistics (featured in Table 2) could be ART 62 “Microbiome, metabolism and genetics of cells”.

#### Ordering of the ARTs based on scoring and maturity

In order to assess the relative anticipated impact of ARTs for GVN transformations, an indicator was developed as follows. For each ART, each GVN adds a special, mostly nonoverlapping perspective. These 20 perspectives were used to form the basic RTI 2018 indicator in the following three steps (see the rows in Table 4). First, each ART’s potential impact on different GVNs was evaluated using the short statements, as described above. Second, for each ART, all potential impact values were summed. Third, the sum potential impact value for each ART was multiplied with the maturity value of each corresponding ART, thus getting *anticipated total value* for each ART.

**Table 4:** Top ten ARTs with highest anticipated value

	Maturity	Passanger transport	Logistics	Manufacturing of goods	Sustenance	Energy supply	Production materials	Built environment	Exchange	Remote impact	Automation of work	Work and income
Neural networks and deep learning	**5**	10	10	5	10	3	3	5	10	5	20	20
Autonomous cars and trucks	**5**	20	20	0	3	5	0	10	10	5	10	5
Environment scanning and positioning	**7**	20	20	3	3	0	0	3	0	5	10	3
AI performing local work on global basis	**4**	10	10	5	5	0	3	3	10	20	5	5
DNA reading and writing (full genome)	**7**	1	3	0	20	0	10	0	3	3	0	0
Rapid development of photovoltaics	**7**	5	5	10	5	20	5	10	1	3	3	3
Commercial platforms for sharing economy	**6**	10	5	0	5	0	0	5	10	20	3	10
Speech recognition/synthesis and interpreting	**6**	3	3	0	3	0	0	3	10	5	5	10
Real time 3D modelling of environment	**6**	20	20	10	5	0	0	5	0	1	5	0
Material scanner — hyperspectral camera	**5**	5	10	5	10	0	5	5	5	5	5	3

	Health care	Redressing disabilities	Aquiring information	Proficiency and its proof	Producing experiences	Safety and security	Collaboration and trust	Existential meaning	Power structures	Sum of potential GVN impacts	Anticipated total value (Sum GVN × Maturity)
Neural networks and deep learning	10	5	20	10	10	5	5	5	20	**191**	**955**
Autonomous cars and trucks	0	10	5	0	5	20	3	3	0	**134**	**670**
Environment scanning and positioning	0	3	10	3	5	5	0	0	0	**93**	**651**
AI performing local work on global basis	10	3	5	20	5	5	10	5	20	**159**	**636**
DNA reading and writing (full genome)	10	5	20	0	0	10	0	5	0	**90**	**630**
Rapid development of photovoltaics	0	0	3	0	0	5	3	3	0	**84**	**588**
Commercial platforms for sharing economy	0	3	3	5	3	0	5	5	5	**97**	**582**
Speech recognition/synthesis and interpreting	3	10	5	5	5	5	10	5	3	**93**	**558**
Real time 3D modelling of environment	0	3	5	0	5	5	0	3	3	**90**	**540**
Material scanner — hyperspectral camera	10	3	10	1	0	10	5	1	5	**103**	**515**

The sum of GVN-specific potential impact points for each ART expresses the anticipated probability of support in R&D and investment communities for advancing the maturation. The strong logarithmic scale of each GVN-specific impact takes into account how R&D spending is not directly related to the size of the first or main potential market but to potential market impacts of an ART on many GVN-based markets. This scoring method assesses generic technologies higher than those with major impact to only a few GVNs; a technology that scores low points in numerous GVNs accumulates more points than one which scores 20 points in only one GVN and no points from most GVNs. This can be easily understood by comparing two examples. The ART “Cloud computing and storage services” got a total score of 399 or 57 without the maturity multiplier while only getting 10 points from the GVN “exchange” and 20 points from none but 1 to 5 points from 13 GVNs. On the other hand, the ART “Curing and preventing dementia” got a total score of only 132 or 44 without the maturity multiplier while getting 20 points from GVN “health”, 10 points from GVN “existential meaning” and just seven 1- to 3-point evaluations [40].

It is important to note that the scoring does not explicitly consider the indirect impact of ARTs to GVNs. For example, VR glasses increase the time spent with immaterial goods, and this might lessen the need for logistics, but this indirect impact is not considered in the GVN of logistics as it does not in this sense satisfy the prime goal or values of the GVN.

The essence of the scoring method is its systematicity. An individual assessment of a specific ART may not be accurate, but it is satisfactory enough to make anticipating the development of the 100 ARTs in relation to each other possible. The order of the ARTs in Table 3 is merely indicative, because of the coarseness of the scoring method.

An important point is that the most transformative ARTs are not necessarily those that are of high economic value but ones that become freely or affordably available. The spreading of Internet access is the best present-day example of such transformative dynamics. In fact, the RTI scoring system is designed with the example of the Internet of 1993 in mind. At that time, the Internet application technology was at a low maturity level, but its envisioned promise was considerable. In 1993, the Internet, when compared to other communication networks of the day, could not be envisioned as critical or strongly transition enabling for any GVN. However, it would most likely have been assigned (1–5) points from the perspective of most GVNs at that time. While 1–3 points would actually have underestimated the impact of the Internet in 20 years in many GVNs, the breadth of the hypothetical potential impact would have resulted in a high overall score.

#### Iteration

Even RTI 2018 is described as a stepwise methodology, the steps should be interpreted as the order of an iterative process where accuracy increases in each iterative round. Initial iterations for the creative formulation of ARTs and GVNs and their qualitative and quantitative interactions have taken place as a part of crowdsourcing and GVN-specific seminars. More formal iterative step has been the critical evaluation of each GVN, including the ART valuations, in 12 stakeholder workshops, two for the overall structure of the RTI 2018 GVN-structure and 10 workshops evaluating each GVN. The 88 expert participants were invited independently by Finnish innovation system organisations. Of the participants, 42 were female and 46 were male.

The stakeholder workshops relied on the ART and GVN definition criteria presented in above subsections. Several improvements were made in GVN definitions at this stage.^4^

### The uses of RTI results

The results of RTI 2018 have been condensed in a report ([40]; in English [41]). The assessment of anticipated total value of 100 ARTs is the core result of that report. However, the report also contains links to crowdsourced evidence behind each ART and analysis of motivations of dominant and challenger regime actor types. For each regime transformation, the results outline the following:

- Potential benefits and threats
- Obstacles
- Opportunities
- Growing professions and skills shortages
- A wide range of policy recommendations (suggested changes in legislation, public funding, education)

The analysis of the challenger regimes yields understanding of how transactions and responsibilities can be structured and what kind of specialisations would be beneficial for transformations. Envisioning the novel means and processes also helps in understanding potential hindrances. There might be missing infrastructure, legislative limitations and missing competences. All of these require policy changes. An example is GVN 12, “Health care and its transformation towards self-diagnostics, gamification of health and individual nutrition”. It is evident that legislative framework, organisational structures and professional capabilities are not optimal for this transformation. It is for the policymakers to decide if they see the anticipated transformation as beneficial and thus the outlined policy measures worth implementing.

The main use of these results relates to various actors’ diverse interests in the future possibilities described in the report. The report has been widely read among educators, R&D departments, ministries and city planners especially in Finland. For example, the City of Helsinki has used the report as a source in long-term city planning [22]. The report has been covered or used as a source in hundreds, mainly Finnish, popular published articles, theses, studies and reports (for example [11], p. 756-757 [26];). Especially, the view upon future skills and professions has been a regular topic in the press. RTI 2018 results have also been used in numerous expert statements in parliamentary committee hearings and the results have been regularly referred to especially in many statements of the Committee for the Future^5^, which originally commissioned the development of the methodology.

Finally, the results have stimulated or supported interaction between actors that are shown to have shared interests in specific challenger regimes or in ARTs with potential to contribute to several regime transformations. For instance, several regional development seminars have gathered actors from companies, universities and universities of applied science. Another example is the Finnish photonics stakeholders’ analysis of the future potential of the field [57], where RTI 2018 results have been referred to multiple times.

No complex future scenario can be relied upon as such as the basis of policies. To increase reliability, RTI includes continuous and transparent reassessment. Both ARTs and anticipated GVN transformations progress are followed critically. This continuous process has already led to some clear changes in ARTs, GVNs and the whole methodology since RTI 2013. A follow-up study ([38], in English [39]) was commissioned by the Parliament of Finland to check how the developments anticipated in 2013 had been realised by 2016, what methodological changes would be useful and what new developments required attention. Despite some clear misses, the results showed that the 25 technologies that scored the highest anticipated value in 2013 had matured on average faster than the rest of the 100, while the second quartile of technologies was on average the second fastest to mature and so forth. As the high anticipated value correlates with ART being relevant in many GVNs, a possible explanation for the rapid advancement might be that the demand for and investments in such ARTs are greater and more stable as it is spread across multiple industries and sectors. This is not investigated empirically, however. A critical follow-up of the 2013 results was included in RTI 2018 both for ARTs and GVN transformations. Topic-specific studies have already partially assessed the RTI 2018 results.

Despite the methodological improvements, the results of RTI 2013 and RTI 2018 have remained sufficiently comparable in the level of the GVNs. They provide a possibility to check how the anticipated transformation of GVNs have advanced in 5 years. Table 5 below shows the GVNs in the order where the challenger regime had advanced the fastest based on the total impact of all 100 ARTs.

**Table 5:** Global value-producing networks (GVNs) are arranged according to the rate of anticipated development speed

Ranking	Value-producing network	The rate with which the anticipated value of GVN has grown [= 10 × LOG (anticipated total value of ARTs in a GVN in 2018/anticipated total value of ARTS in a GVN in 2013)]
1	Passenger transport	6.7
2	Logistics	6.3
3	Work and income	5.9
4	Automation of work	5.8
5	Sustenance	5.3
6	Manufacturing of goods	5.2
7	Built environment	5.0
8	Exchange	4.6
9	Acquiring information	4.1
10	Safety and security	4.1
11	Remote impact	4.0
12	Existential meaning	3.7
13	Power structures	3.1
14	Producing experiences	2.7
15	Health care	2.6
16	Materials	2.4
17	Energy supply	2.3
18	Collaboration and trust	2.3
19	Proficiency and its proof	1.0
20	Redressing disabilities	0.8

The challenger regimes seem to advance the fastest in the domains of transportation, work and income, automation of work, nutrition, manufacturing, construction and exchange. Here, the relevant ARTs appear to have rather powerful support, and real-life embryonic transformation is already on the way. From a policy perspective, the relevant question is whether one wants to accelerate the transformation by removing its structural barriers related to, for example standards, regulations or legislation — or whether one wants to slow the transformation down by safeguarding the interests of the proponents of the dominant regime.

The comparison also shows that transformation had advanced less in the GVNs at the bottom of the list: proficiency and its proof (education and proving of learning) and redressing disabilities (technological assistance to disabled people). This is not necessarily because of the lack of radical technologies but instead due to the strong reification of power structures in the dominant regime (esp. in education^6^) and lack of substantial investment (esp. for technologies for the disabled). Here, the relevant ARTs seem to lack powerful proponents, which means that more active policy support in addition to removal of structural barriers would most likely be needed, should the decision-makers see benefits in the envisioned transformations.

Another type of use of RTI relates to follow-up studies that build on the methodology of RTI 2018. This also functions as additional iteration as all these studies gather new evidence and insight in the same framework. The Development of Genetic Engineering in Different Areas of Application 2018–2020 [42] was initiated as a reaction to EU Commission’s gathering of information of novel genomic techniques from the member states. In Finland, a related survey was coordinated by the Board for Gene Technology. The board approached the Committee for the Future in the Parliament of Finland, asking if the committee had deliberated on the state of gene technologies since 2018. RTI 2018 was utilised as a framework updating info on relevant ARTs and actor motivation. *Pandemiateknologiat* (“Pandemic technologies”) [43] was commissioned by the Committee for the Future to understand how technology could better be utilised and regulated in order to face future pandemics. It used directly the RTI 2018 GVN framework for analysing how different technologies were being used to prevent or mitigate the ill effects of the COVID-19 pandemic. That report also evaluated how the pandemic influenced the anticipated transformations towards the challenger regimes and provided several policy recommendations for appropriate GVNs. The article in *Digital Innovation and the Future of Work* [45] followed more closely the GVN structure of the original RTI 2018 report and described an overview on how each anticipated GVN transformation would change work.

The *Towards a Better Future* [44], in turn, showed how the RTI can be used for assessing means to achieve societal goals. In terms of Porter’s (2005) typology, RTI 2018 is explorative, whereas *Towards a Better Future* has a normative motivation. In Towards a better future, the role of RTI 2018 is to provide holistic regime transformation visions whose credibility as sustainability goals is then evaluated. The GVN transformations are first described as if each challenger regime would become well-regulated mainstream without major problems. Then, these are evaluated with respect to each United Nations’ Agenda 2030 sustainable development goals (SDGs), using a qualitative questionnaire assessing how a transformation supports each SDG’s subgoals and what potential threats and tensions that would entail. The report builds on the premise that goal-oriented evaluation only becomes possible when holistic regime transformations are considered. If taken out of their socio-technical context, the sustainability impacts of technologies cannot be evaluated. In other words, the real question is not what technologies we have but how we use what we have. The policy argument is that emphasis should be given to issues where societal intervention could diminish threats and help turn opportunities into positive development. Attention should also be paid to potential conflicts between various sustainability goals. This is necessarily a qualitative assessment.

## Discussion

The RTI methodology has at least four limitations, to be discussed here. The first is the strong role of two authors of this article in the qualitative and quantitative assessment of the impact of ARTs. The alternative would be to have an expert panel for each GVN (or for each group of ARTs) and have those experts do the final scoring, which would resemble the route taken in the TechCast project, for example [21]. The challenge in both alternatives is that the scoring needs to be balanced between GVNs and groups of ARTs, so that, e.g. score 5 means the same thing across sectors. As such experts do not exist that would be readily familiar with technological opportunities across all sectors, the expert panels would have to spend unrealistic amounts of time familiarising themselves with new socio-technical contexts. In RTI, it has been considered more feasible for two people to balance the final scoring in a somewhat crude manner, adhering to the defined criteria and iterating between the varied discussions and arguments in expert panels and in the social media group. Even this is somewhat time-consuming, which can be considered a practical drawback of the RTI tool.^7^

The second limitation of the RTI is that the maturity of ARTs has not been defined in a GVN-specific manner. In reality, novel technologies tend to resolve technological bottlenecks in an economically feasible manner earlier in one application context than another. For example, augmented reality glasses (ART 19) can be considered nearing maturity for remote impact (GVN 9) in maintenance work but due to high cost, bulky form and missing applications, but much less mature for producing experiences (GVN 16). A GVN-specific maturity scale would possibly increase accuracy of the evaluations, but due to resource limitations, a single maturity value for each ART was selected. This practice can be defended as the funding of the ART development in one GVN can generally be seen to increase its maturity for other GVNs too.

The third limitation is that RTI 2018 does not discuss how ARTs and GVNs develop step by step in their mutual interaction during 2018–2037. In other words, it does not recognise path dependencies that might occur between the beginning and the end states of the anticipated period. This limitation is partially bypassed by defining ARTs as bundles of alternative solutions instead of separate technologies. As a consequence, winning technologies cannot be anticipated even when the maturation of ARTs is. This is not necessarily a big problem from the perspective of needed regulation, but it may be an issue from the point of view of credibility of links between ARTs and GVNs and concerning the urgency of investments in different technologies.

**Fig. 4:**
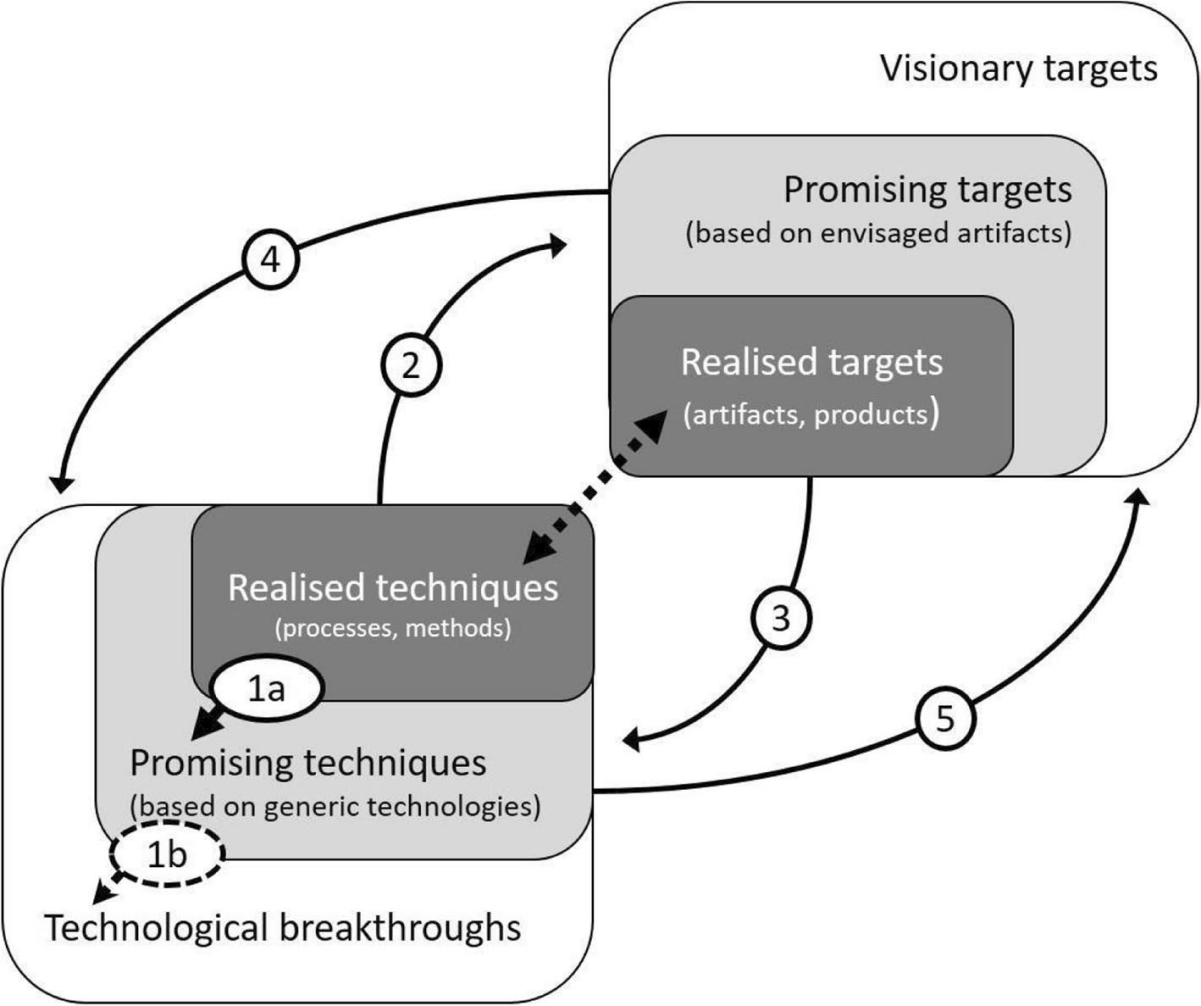
Technology generalisations as interaction processes of technology push and demand pull (modified from [19])

Osmo Kuusi and Martin Meyer have developed an approach or model that might help new versions of RTI to overcome this limitation [19, 27, 30, 31]. Kuusi and Meyer have called the maturation process of a technical niche innovation together with its applications “technology generalisation process”. The term stresses anticipation-related learning processes that continuously change technological innovations in interaction processes of technology push and demand pull (Fig. 4).

In Fig. 4, arrows illustrate different kinds of technology generalisations. Using the model, Kuusi and Meyer have analysed the future prospects of niche innovations, e.g. carbon nanotubes and cellulosic ethanol based on patent applications [19, 31].

The arrows of the Fig. 4 can be given the following interpretations that connect them to interaction processes between developing ARTs and GVNs. It is possible to divide expected or anticipated technology generalisations into three basic types. First, some generalisations combine made technical discoveries related to an ART *to new discoveries* related to the same ART (arrow 1 a & b); the second class of generalisations connect made discoveries related to an ART *to new applications* in emerging GVNs (2 & 5). The third group refers to generalisations where an ART is *replaced or improved by* another ART in realising of an anticipated feature of some GVN (3 & 4). The 1a, 2 and 3 represent earlier generalisations (e.g. during the first 10 years of the 20 years’ anticipation horizon) that are needed before 1b, 5 and 4 are possible (later, e.g. during the second 10 years). One could call 1a, 2 and 3 *expected* generalisations and 1b, 5 and 4 *anticipated* generalisations.

The fourth limitation concerns the holism of the RTI methodology. Inevitably, it can only be accomplished in a coarse manner and by simplifying methodological choices. These are needed especially concerning the fixed prime goals of GVNs. For further research, an important challenge is to define the quality criteria more explicitly for anticipated GVNs and their prime goals in particular. In this paper, the quality of defining GVNs has been promoted by considering transparency, consistency and simplicity as boundary conditions. The further highly important and difficult boundary condition concerns how the 20 GVNs cover all relevant social functions in 2037. Our choice has been to link the targets of GVNs to eight social functions of Stiglitz et al. [63]. They function as a kind of checklist of the coverage of 20 GVNs. The existing public, private and third sector organisations have also been assessed to check that there is no meaningful residual that did not find a place within the 20 GVNs. There is however a big basic problem in this kind of checking. Relevant social functions depend beside human needs also on available means or technologies. In the distant future, the colonisation of Mars might be an important social function though its role as a social function is presently very limited. Changing human interests^8^ are based on human needs and available means, and thus, it is important to realise that the relative importance of GVNs will change during the next 20 years, and it might be necessary to construct a new GVN or remove some of the existing ones.

As a final point in this discussion, the authors would like to bring up that back in 2013; when the development of RTI was initiated, many of the envisioned 20 GVN developments were hypothetical. The facts that key ARTs in all 20 GVNs had increased their maturity by 2016 and a plausible challenger regime with advancing ARTs could be defined for all GVNs in 2018, it took the authors by somewhat of a surprise. While interesting, this was also alarming; if radically transformative change is to be expected on such a broad scale, will societal institutions and infrastructure be able to keep up with it? Will, for example, a large part of the existing auto industry collapse due to too slow adaptation speed? Will the education systems of different countries be able to provide continuous education with the same speed as existing competences become outdated? Is the reason behind Russian military aggression in Ukraine at least partially due to the anticipated energy sector transformation? Should we remind ourselves of Toffler’s *Future Shock* [65] or the analysis of long cycles following Kondratjev’s and Schumpeter’s work [24, 61, 67], which seemed to show that each radical transformation is preceded by major political, military or economic crises?

The motivation behind this paper is partly backed up by this alarm. The methodology should indeed be critically scrutinised because of the broad scope of changes claimed to be anticipated with it. The results further indicate that there is an urgent need for further methodologies and practical processes through which decision-makers could become familiar with anticipatory thinking and in particular anticipatory thinking related to impacts of novel technologies on existing socio-technical structures.

## Conclusions

This paper introduces a methodology called Radical Technology Inquirer (RTI). The tool is designed to provide results that enable decision-makers to anticipate long-term socio-technical changes created by novel technologies. Such anticipation serves the creation of policies, regulation, legislation, infrastructure and other capabilities that seek to support positive developments and avoid negative consequences. The methodical steps of RTI are presented in sufficient detail to allow replication.

The authors claim that existing technology foresight approaches do not enable fulfilling the above aim. Most technology foresight approaches serve other needs: technology diffusion models are useful for economists, and roadmapping approaches serve strategy work in organisations. These perspectives work poorly when there is a need to understand the cumulative societal impact of multiple kinds of technologies. Even when the scope and the time horizon of the foresight approach are quite similar with RTI, as is the case with the TechCast project, the focus on development trajectories of single technologies does not help much in the key objective RTI, which is to help public decision-making concerning regulation and strategic investments in relation to broader socio-technical developments.

The authors propose that the holistic interpretation of technology foresight serves better the decision-making needs presented in this paper than TechCast’s expert evaluations of the future development of single technologies. The authors consider that RTI is able to support proper regulation and public and private strategic investments focusing on three complementary facets to systemic foresight suggested by Dufva et al. [9]: knowledge creation and diffusion as the whole picture of 20 global value-producing networks (GVNs) and 100 emerging anticipated radical technologies (ARTs), enhancing relations and networking of various stakeholders that can use the RTI results to identify and discuss joint interests especially in relation to challenger regimes, and suggesting needed capabilities based on 20 GVNs.

## Data Availability

Internet sources of the data based on which recent anticipated radical technologies (ARTs) are constructed are available in the descriptions of the 100 ARTs in the last basic report of the RTI [41]. The access to social media support group where new evidence of radical technologies of the ARTs has been and is being added and evaluated (not in English) can be requested from the corresponding author.

## Abbreviations

RTI: Radical Technology Inquirer
GVN: Global value-producing networks
ART: Anticipated radical technology
MLP: Multi-level perspective
AI: Artificial intelligence
RIBRI: Radical Innovation Breakthrough Inquirer

## Footnotes

1 https://enspire.science/trl-scale-horizon-europe-erc-explained/

2 Instead of needs that relate to prime goals, in this paper the wider, actor and means related concept “interest” is used. From this perspective, the targets that people try to achieve or artefacts that they demand are combinations of perceived needs and available means. Interest is a concept that combines needs and considerations concerning available means, e.g. available technologies (compare [27]).

3 In the original 2018 report, the share/GDP was expressed with concrete sums that matched the scale of Finnish economy.

4 For example, for most GVNs, there was a strong consensus of the form of the potential challenger regime. Multiple credible challenger scenarios emerged only for the GVN of sustenance; instead of localised food production and robotized preparation, development might become directed towards the full robotisation of countryside farming. Localised challenger regime was chosen as it more clearly deviates from the dominant regime. A somewhat less credible yet possible radical scenario could be formed around the advancement of technologies that preserve packaged fresh food for a long time at room temperature. The collapsing of the cost of transport and long-term storage of perishable foodstuff would strongly undermine the relative cost-effectiveness of local scale and in many places also the national scale of food production.

5 It is to be noted that the second and fourth authors have been involved in drafting these committee statements, one in the role of senior advisor of the committee one as a member of Parliament presenting the policy problems. The first and third authors have designed the key methodological elements of RTI 2018. The second and fourth authors have been involved in applying the findings into committee’s work and in drafting the present paper.

6 During the review rounds of this paper, the power structures of the education GVN were shifting, however, as distant learning was suddenly made necessary by the rapidly spreading COVID-19. The consequences of the crisis in Ukraine, in turn, appear to accelerate transformation in the GVN of energy supply.

7 The use of artificial intelligence has sometimes been suggested as a possible solution, but it has been rejected by the authors on the basis that AI would be weak in understanding social needs/functions, let alone imagining how these could be satisfied by novel means that do not yet fully exist.

8 About the concept of interest in futures research, see Kuusi and Hautamäki [29].
